# Supplementary material for: The lack of K13-propeller mutations associated with artemisinin resistance in Plasmodium falciparum in Democratic Republic of Congo (DRC)
Source: PLoS One. 2020 Aug 21;15(8):e0237791. doi: 10.1371/journal.pone.0237791 (PMC7446852; doi:10.1371/journal.pone.0237791)
Supplement: Attachment — Submitted filename: Responses to Reviewers.docx [file pone.0237791.s003.docx]

**Responses to Reviewers**

**Reviewer #1:**

***We thank the reviewer very much for the comments, which helped us improving the manuscript.***
- Tile: Consider to revise the title and running title to cover the whole picture of the study. Eg, Lack of K13….. in Congo. Running title: Pf K13 polymorphism in DROC.

A/ ***Thank you for the suggestion. We’ve revised the title and running title.***

- Line 43, M472I, V534A and A569T are not new mutation. These are already reported. (DOI: 10.9734/JAMMR/2017/34192) <https://www.ncbi.nlm.nih.gov/pmc/articles/PMC4955562/> (Major comment) If you want to say these are new SNPs detected in DRC, clearly mention in abstract also.

A/ ***Thank you for pointing this out. We’ve corrected in the manuscript: we’ve removed the term "new" and preferred say "previously detected and not yet detected in DRC"***

- The author use the term "novel mutation" for SNP detected that were not reported before in DRC. But these SNPs are already reported in other areas. It means it is not novel mutation. Correct the term used in the paper. (Figure also for "new mutation")

A/ ***Thank you for the remark. We’ve corrected in the whole text.***

- In discussion, among the previous reported SNP, number as well as percentage should be shown.

A/ ***Thank you for the suggestion. We’ve added number and percentage for previous reported SNP.***

- The authors also used the term "New developed pfk13 PCR" for newly designed primer. It is also not need to mention the term "new developed" that is for new innovative procedures that is not reported elsewhere.

A/ ***Thank you for the remark. We’ve removed the term* "New"**

- Here again, why the authors do not use the previously published primers set? Describe it. Moreover, the primers used in this study cover only codons 427 – 595. So, it will miss the SNP beyond the aa 595 in K13 propeller gene. A676 and H719 were also interesting SNPs and reported. The authors need to describe the rationale for it.

A/ ***Thank you for the comment. We’ve designed new primers to amplify a segment containing all validated mutations and majority of candidate mutations associated to artemisinin resistance. We’ve taken into account the limit size (700-800bp) of segment to ensure a better quality of sequences obtained from Sanger sequencing method used in this study. A broader view would have been desirable to explore other candidate mutations. We’ve mentioned this limitation in discussion section.***

- It is better to include the basic demographic character of the participants as well as the clinical data of day3 positivity (if possible)

A/ ***Thank you for the suggestion. We’ve included in the results the repartition of participants per age range and per sex. Clinical follow-up data were not assessed by the study.***

- Describe the first line and second line treatment for pf in Congo in Discussion.

A/ ***Thank you for the suggestion. We’ve include an overview of first-line and second-line treatment used in DRC for uncomplicated malaria in the discussion section.***

**Reviewer #2:**

1. Summary of the research
Yobi [et.al](http://et.al/) have looked at polymorphisms in the Kelch 13 propeller gene in P.falciparum isolates from clinical samples in the DRC and have reported both existing and new non-synonymous mutations in the gene from the region; although none of these mutations have been directly implicated in ART resistance. The study has been conducted with comprehensive coverage of the major malaria endemic regions of the country with an adequate sample size. The diagnostics and methodology are robust and the results are expressed in a logical manner. Given the fact that artemisinin is the mainstay for treatment for uncomplicated falciparum malaria, monitoring its activity is important in the field and in this regard, the authors have looked into K-13 mutations which is a preliminary molecular marker for possible artemisinin resistance.

***We sincerely thank the reviewer for these very encouraging comments and remarks to improve this work.***

2. Examples and evidence

Major issues

1. The authors have looked into K-13 polymorphisms within a limited codon range (427-595). Although most of the validated K-13 propeller mutations associated with ART resistance are covered in this range, a broader scope could have been adopted to cover other candidate mutations. Since ART resistance is still evolving in Africa, a comprehensive investigation into all established and probable markers of ART resistance would have given a clearer picture of the situation.
***A/ Thank you for the comment***. ***As you said***, ***we’ve amplified a segment containing all validated mutations and majority of candidate mutations associated to artemisinin resistance. We’ve taken into account the limit size (700-800bp) of segment to ensure a better quality of sequences obtained from Sanger sequencing method used in this study. A broader view would have been desirable to explore other candidate mutations. We’ve mentioned this limitation in discussion section.***

2. The authors have used a Taqman based qPCR assay for P.falciparum detection. As such, it would be good if qPCR based parasite quantification data is mentioned in the results. It would be especially interesting to learn about the difference and correlation (if any) in the parasite counts in individuals harbouring wild-type versus mutant K-13 isolates.
A/ ***Thank you for the suggestion. In this study, we did not quantify parasite densities in participants, we’ve just noted the presence or not of the Plasmodium falciparum. We will take this suggestion into account in our future studies.***

3. No mention is made of the patient demographics in the manuscript. Any possible association between patient demographics and isolation of mutant parasite strains should be looked into and included in the results.
***A/Thank you for the suggestion. We’ve included in results the distribution of patients per age range and per sex and the distribution of mutations per age range of patients.***

Minor issues

1. Since drug resistance mutations often have a tendency to cluster together, it would be good to know if the isolates harbouring K-13 mutations also have other antimalarial drug mutations. If these isolates have been subjected to such tests, the information should be included in the results.
***A/ Thank you for pointing this out. Other antimalarial drug mutations including mutations on pfcrt gene associated with chloroquine resistance were addressed in another paper.***

2. The sequenced isolates do not mention any accession numbers. If these sequences have not been deposited in any data repository, the information should be included in the methodology.

***A/ Thank you for pointing this out. We’ve mentioned in Methods section the sequences were not deposited in any data repository.***
