# Supplementary material for: The lack of K13-propeller mutations associated with artemisinin resistance in Plasmodium falciparum in Democratic Republic of Congo (DRC)
Source: PLoS One. 2020 Aug 21;15(8):e0237791. doi: 10.1371/journal.pone.0237791 (PMC7446852; doi:10.1371/journal.pone.0237791)
Supplement: Attachment — Submitted filename: Responses to Reviewer.docx [file pone.0237791.s004.docx]

**Responses to Reviewers**

**Reviewer #1:**

***We sincerely thank the reviewer for the suggestion, raised to improve the quality of the manuscript.***

Q/ Table 1. Efficacy should be "frequency or number of the cases" If you have no hypothesis on ART based on sex, you should not compare between male and female. If you have hypothesis, you can compare and statistical analysis will be needed. For my opinion, you can simply describe basic demographic character of the participants (eg Age mean(SD), M:F, region etc only

***A/ Based on the suggestion by the reviewer, we’ve opted to describe basic demographic characters of the participants (median age, sex ratio) only in the text of the manuscript in place of the table. The distribution of K13 polymorphim per region (site) of participants can be found in table 1 of the newly revised version.***
